# Supplementary material for: Optimal gap-affine alignment in O(s) space
Source: Bioinformatics. 2023 Feb 7;39(2):btad074. doi: 10.1093/bioinformatics/btad074 (PMC9940620; doi:10.1093/bioinformatics/btad074)
Supplement: btad074_Supplementary_Data [file btad074_supplementary_data.pdf]

Supplementary material for Optimal gap-affine alignment in $O(s)$ space

Santiago Marco-Sola, Jordan M. Eizenga, Andrea Guarracino, Benedict Paten, Erik Garrison, and Miquel Moreto

1 Proof of the correctness lemma

In order to reason about the properties of the WFA dynamic programming structures, it is helpful to invoke certain properties of the Needleman-Wunsch dynamic programming matrices. Accordingly, we will provide the recursions here to introduce the notation.

$$\begin{aligned} D_{i,j} &= \min\{M_{i-1,j} + o + e, D_{i-1,j} + e\} \\ I_{i,j} &= \min\{M_{i,j-1} + o + e, I_{i,j-1} + e\} \\ M_{i,j} &= \min\{I_{i,j}, D_{i,j}, M_{i-1,j-1} + x \cdot \mathbb{I}(q[i-1] \neq t[j-1])\}, \end{aligned} \tag{1}$$

where $\mathbb{I}$ is the indicator function that evaluates to 1 if its argument is true and 0 otherwise. The base case of the recursion is $M_{0,0} = 0$. We also adopt the convention that $D_{0,j} = I_{i,0} = \infty$ for all i and j. An optimal alignment can be identified with a *traceback path* through these matrices: a sequence of cells that indicate which of the options from the recursion achieved the minimum score.

Before proving the correctness lemma, we prove two useful properties of the Needleman-Wunsch matrices.

Lemma 1. *M is monotonically non-decreasing along each diagonal.*

Proof. Choose integers i and j such that $0 \leq i < m$ and $0 \leq j < n$, and we will show that $M_{i,j} \leq M_{i+1,j+1}$, which is sufficient to prove the claim. $M_{i+1,j+1}$ corresponds to the score of an optimal alignment of $q_{0:i}$ and $t_{0:j}$. Any traceback path of this alignment must include a coordinate (i, y) with $y \leq j$ or (x, j) with $x \leq i$. Without loss of generality, assume that there is an optimal alignment path that includes (i, y), and choose y to be the maximal such value within this path. We consider two cases:

1. $y = j$. Then (i, j) is on the traceback path from $M_{i+1,j+1}$ and hence $M_{i,j} \leq M_{i+1,j+1}$.
2. $y < j$. Then there must be at least $j-y$ horizontal transitions on the traceback path following (i, y) for it to end in diagonal $i - j$. Moreover, since y is chosen to be maximal, $(i, y + 1)$ is not on the traceback path, and there must therefore be at least one gap opened after (i, y). This implies $M_{i+1,j+1} \geq M_{i,y} + o + (j - y)e$. We also have $M_{i,j} \leq M_{i,y} + o + (j - y)e$, since it is possible to reach (i, j) by taking $j - y$ horizontal transitions starting from (i, y).

□

Lemma 2. *D and I are monotonically non-decreasing along each diagonal, excluding the boundaries $D_{0,\cdot}$ and $I_{\cdot,0}$.*

Proof. The proofs for I and D are essentially identical, so we will prove the claim only for I. The argument will be proved by induction on decreasing values for the diagonal k. The base case $k = m - 1$ is trivially true because there is only one cell in I in this diagonal (excluding the boundary). Consider i and j such that $0 \le i < m$ and $0 < j < n$, and assume that the induction hypothesis holds for all diagonals $k > i - j$. We will show that $I_{i,j} \le I_{i+1,j+1}$, which is sufficient prove the induction claim for $k = i - j$. Consider two cases.

1. $I_{i+1,j+1} = M_{i+1,j} + o + e$. Then, by Lemma 1, we have
$$I_{i,j} \le M_{i,j-1} + o + e \le M_{i+1,j} + o + e = I_{i+1,j+1}. \tag{2}$$
2. $I_{i+1,j+1} = I_{i+1,j} + e$. Then, by the induction hypothesis, we have
$$I_{i,j} \le I_{i,j-1} + e \le I_{i+1,j} + e = I_{i+1,j+1}. \tag{3}$$

□

We are now equipped to prove the central lemma that demonstrates correctness.

Lemma 2.1 (from main text). *The optimal alignment score $s_{opt} \le s$ if and only if there exist s_f, s_r, and k such that $|s_f - s_r| \le p$ and at least one of the following is true:*

1. $s_f + s_r = s$ *and* $\overrightarrow{\mathcal{M}}_{k,s_f} \ge \overleftarrow{\mathcal{M}}_{k,s_r}$
2. $s_f + s_r = s + o$ *and* $\overrightarrow{\mathcal{I}}_{k,s_f} \ge \overleftarrow{\mathcal{I}}_{k,s_r}$
3. $s_f + s_r = s + o$ *and* $\overrightarrow{\mathcal{D}}_{k,s_f} \ge \overleftarrow{\mathcal{D}}_{k,s_r}$,

and further, $\overleftarrow{\mathcal{M}}_{k,s_r}$ (resp. $\overleftarrow{\mathcal{I}}_{k,s_r}$, $\overleftarrow{\mathcal{D}}_{k,s_r}$) is included in the traceback of an alignment with score at most s if the first (resp. second, third) condition is true.

Proof. ($\Rightarrow$) Let (i, j) be a coordinate along some optimal traceback path where the dynamic programming value has the minimum difference from $s_{opt}/2$. If there are ties, choose the first among the coordinates that achieve the minimum. We consider three exhaustive cases. In each of them, our goal will be to produce the values s_f, s_r, and k as required by the claim.

1. *The path is in M at (i, j).* Then the path up to (i, j) and the path after (i, j) correspond to partial alignments in the forward and reverse direction respectively, and their scores are $s_f = M_{i,j}$ and $s_r = s_{opt} - M_{i,j}$. Taking $k = i - j$, we know that the f.r. points in the k-th diagonal must be at least as far as this coordinate in their respective directions: $\overrightarrow{\mathcal{M}}_{k,s_f} \ge i \ge \overleftarrow{\mathcal{M}}_{k,s_r}$.

   Because adjacent positions in an optimal traceback path can differ by at most p, we have both $|s_f - s_{opt}/2| \le p/2$ and $|s_r - s_{opt}/2| \le p/2$. These imply $|s_f - s_r| \le p$ by the triangle inequality.
2. *The path is in I at (i, j) and not also in M at (i, j).* Then (i, j) is part of a gap that begins at (i, j') for some $j' < j$ and ends at $(i, j' + \ell)$ where $j' + \ell > j$, else the path is also in M at (i, j). Consider the quantity $x = (s_{opt} - 2M_{i,j'})/2e$ across three cases.

   2.1. $x \le 1/2$. Let $s_f = M_{i,j'}$ and $s_r = s_{opt} - M_{i,j'} + o$. These correspond to the scores of the partial alignments before and after (i, j'), respectively. Therefore we take $k = i - j'$, and, as previously, the f.r. points within this diagonal must obey the inequality $\overrightarrow{\mathcal{M}}_{k,s_f} \ge i \ge \overleftarrow{\mathcal{M}}_{k,s_r}$.
   Note that $M_{i,j'} \le s_{opt}/2$ else $I_{i,j'}$ would not achieve the minimum difference from $s_{opt}/2$. This implies $x \ge 0$, and in particular $|x| \le 1/2$. Therefore,
   $$|s_f - s_r| = |s_{opt} - 2M_{i,j'} + o| \le |o + 2ex| \le o + 2e|x| \le o + e \le p. \tag{4}$$

2.2. $1/2 < x < \ell - 1/2$. Let x^* be the nearest integer to x, and let $s_f = I_{i,j'+x^*}$ and $s_r = s_{opt} - I_{i,j'+x^*} + o$. These correspond to the scores of the partial alignments before and after $(i, j' + x^*)$, respectively. Therefore we take $k = i - j' - x^*$, and, as previously, the f.r. points within this diagonal must obey the inequality $\overrightarrow{\mathcal{I}}_{k,s_f} \geq i \geq \overleftarrow{\mathcal{I}}_{k,s_r}$.

Noting that $|x - x^*| \leq 1/2$ by construction, we also have

$$|s_f - s_r| = |s_{opt} - 2M_{i,j'} - 2x^*e| \leq 2e|x - x^*| \leq e \leq p. \quad (5)$$

2.3. $x \geq \ell - 1/2$. Let $s_f = I_{i,j'+\ell}$ and $s_r = s_{opt} - I_{i,j'+\ell} + o$. These correspond to the scores of the partial alignments before and after $(i, j' + \ell)$, respectively. Therefore we take $k = i - j' - \ell$, and, as previously, the f.r. points within this diagonal must obey the inequality $\overrightarrow{\mathcal{I}}_{k,s_f} \geq i \geq \overleftarrow{\mathcal{I}}_{k,s_r}$.

Noting that $s_{opt}/2 \leq I_{i,j'+\ell}$ else $j \geq j' + \ell$, and also that $I_{i,j'+\ell} = M_{i,j'} + o + \ell e$, we can obtain

$$\begin{aligned} s_{opt} &\leq 2M_{i,j'} + 2o + 2\ell e \\ s_{opt} - M_{i,j'} - \ell e &\leq M_{i,j'} + 2o + \ell e \\ s_r &\leq s_f + o. \end{aligned} \quad (6)$$

Since $x \geq \ell - 1/2$, we also have

$$\begin{aligned} s_{opt} - 2M_{i,j'} &\geq (2\ell - 1)e \\ s_{opt} - M_{i,j'} - \ell e &\geq M_{i,j'} + (\ell - 1)e \\ s_r &\geq s_f - o - e. \end{aligned} \quad (7)$$

These together imply $|s_f - s_r| \leq o + e \leq p$.

3. *The path is in D at (i, j) and not also in M at (i, j).* Same as the previous case.

($\Leftarrow$) We consider the three conditions separately.

1. Let (i_1, j_1) be the coordinates in M corresponding to $\overrightarrow{\mathcal{M}}_{k,s_f}$ and likewise (i_2, j_2) for $\overleftarrow{\mathcal{M}}_{k,s_r}$. The partial alignments corresponding M_{i_2,j_2} and $\overleftarrow{\mathcal{M}}_{k,s_r}$ can be concatenated into a full alignment with score $M_{i_2,j_2} + s_r$. By Lemma 1, this score is at most $M_{i_1,j_1} + s_r = s_f + s_r = s$.

2. Let (i_1, j_1) be the coordinates in I corresponding to $\overrightarrow{\mathcal{I}}_{k,s_f}$ and likewise (i_2, j_2) for $\overleftarrow{\mathcal{I}}_{k,s_r}$. The partial alignments corresponding I_{i_2,j_2} and $\overleftarrow{\mathcal{I}}_{k,s_r}$ can be concatenated into a full alignment with score $I_{i_2,j_2} + s_r - o$. By Lemma 2, this score is at most $I_{i_1,j_1} + s_r - o = s_f + s_r - o = s$.

3. Same as the previous condition.

□

2 Complementary evaluation on simulated data (short sequences)

Time (ms)

	100 bp						1 Kbp						10 Kbp					
	**0.1%**	**1%**	**5%**	**10%**	**20%**	**40%**	**0.1%**	**1%**	**5%**	**10%**	**20%**	**40%**	**0.1%**	**1%**	**5%**	**10%**	**20%**	**40%**
edlib	115	116	117	120	123	126	99	102	103	122	144	179	273	314	361	431	575	848
bitpal	25	24	24	24	24	25	128	128	130	129	130	132	1240	1238	1241	1252	1249	1247
ksw2-extz2	127	128	148	163	174	176	887	890	897	908	912	917	9903	9862	9821	9830	9897	9853
WFA-high	5	5	29	60	125	237	1	8	73	207	547	1311	1	28	612	1991	5664	13265
WFA-med	7	7	43	113	288	626	1	15	219	688	2026	4802	2	91	1893	6627	20205	47362
WFA-low	7	7	42	132	345	752	1	17	260	830	2429	5744	2	110	2294	7957	24080	56184
wfalm	9	9	34	79	195	449	2	12	162	587	1730	4282	3	81	1797	6447	19181	45360
wfalm-low	11	11	50	128	328	746	3	18	286	943	2828	6793	4	130	2789	10069	30354	76382
wfalm-rec	9	9	91	163	455	1118	3	23	476	1706	5458	13730	4	236	6112	22254	70693	187436
BiWFA	10	10	48	97	188	339	3	19	120	391	937	2145	3	53	774	2446	6911	15764
BiWFA-score	11	11	50	92	165	278	2	12	73	196	438	939	3	26	337	1094	3138	7464

Memory (MB)

	100 bp						1 Kbp						10 Kbp					
	**0.1%**	**1%**	**5%**	**10%**	**20%**	**40%**	**0.1%**	**1%**	**5%**	**10%**	**20%**	**40%**	**0.1%**	**1%**	**5%**	**10%**	**20%**	**40%**
edlib	4	4	4	4	4	4	4	4	4	5	4	4	4	4	4	4	4	4
bitpal	4	4	4	4	4	4	4	4	4	4	4	4	4	4	4	4	4	4
ksw2-extz2	4	4	4	4	4	4	6	6	6	6	6	6	193	193	195	193	196	195
WFA-high	6	4	4	6	5	5	4	5	7	8	15	28	8	14	56	128	313	714
WFA-med	4	6	6	4	4	4	4	4	4	4	5	9	6	5	16	35	81	176
WFA-low	4	4	4	4	4	4	4	4	4	4	5	9	4	5	13	25	60	126
wfalm	4	4	4	4	4	4	4	4	4	4	5	8	4	5	19	54	148	347
wfalm-low	4	4	4	4	4	4	4	4	4	4	4	5	4	4	7	10	16	35
wfalm-rec	4	4	4	4	4	4	4	4	4	4	4	4	4	4	5	6	7	9
BiWFA	3	3	3	3	4	4	4	3	4	6	5	5	7	6	6	6	5	13
BiWFA-score	2	2	2	2	2	3	2	2	3	2	2	3	3	3	3	3	4	8

Table S1: Execution time (ms) and memory (MB) required per 1M bases aligned, using simulated sequences (100bp to 10Kbp).

3 Complementary evaluation on simulated data (long sequences)

Time (seconds)

	**100 Kbp**						**1 Mbp**						**2 Mbp**					
	**0.1%**	**1%**	**5%**	**10%**	**20%**	**40%**	**0.1%**	**1%**	**5%**	**10%**	**20%**	**40%**	**0.1%**	**1%**	**5%**	**10%**	**20%**	**40%**
edlib	0.46	0.6	1	2	4	5	0.7	2	9	17	35	67	0.9	4	18	35	69	135
bitpal	12.3	12.3	12	12	12	12	122.9	123	122	123	123	124	247.3	249	249	248	247	248
ksw2-extz2	97.6	97.0	97	96	97	96	n/a	n/a	n/a	n/a	n/a	n/a	n/a	n/a	n/a	n/a	n/a	n/a
WFA-high	0.01	0.4	8	28	84	203	0.1	4	84	312	n/a	n/a	0.1	8	n/a	n/a	n/a	n/a
WFA-med	0.01	0.9	25	89	272	576	0.1	12	457	1922	3690	6594	0.2	31	n/a	n/a	n/a	n/a
WFA-low	0.02	1.1	26	101	301	635	0.1	14	901	4394	4857	7389	0.2	43	4670	7710	9813	14910
wfalm	0.02	1.3	27	90	268	646	0.2	12	255	841	n/a	n/a	0.3	25	n/a	n/a	n/a	n/a
wfalm-low	0.02	2.0	48	164	494	1208	0.2	23	466	1525	4418	10605	0.5	45	893	2990	8779	21147
wfalm-rec	0.04	4.6	127	447	1402	3522	0.6	66	1618	5792	17752	44669	1.3	143	3421	11979	37747	n/a
BiWFA	0.02	0.4	6	20	61	147	0.1	3	61	218	680	1701	0.1	6	130	466	1429	3501
BiWFA-score	0.01	0.2	3	10	30	73	0.0	1	30	112	355	894	0.0	3	67	245	750	1791

Memory (MB)

	**100 Kbp**						**1 Mbp**						**2 Mbp**					
	**0.1%**	**1%**	**5%**	**10%**	**20%**	**40%**	**0.1%**	**1%**	**5%**	**10%**	**20%**	**40%**	**0.1%**	**1%**	**5%**	**10%**	**20%**	**40%**
edlib	5	5	5	5	5	5	13	13	13	13	13	13	22	23	23	22	23	23
bitpal	6	4	4	4	6	6	10	10	10	10	10	10	14	12	17	15	13	14
ksw2-extz2	19081	19083	19067	19081	19083	19047	n/a	n/a	n/a	n/a	n/a	n/a	n/a	n/a	n/a	n/a	n/a	n/a
WFA-high	10	131	2707	8981	26667	65123	149	12505	269893	932199	n/a	n/a	537	49356	n/a	n/a	n/a	n/a
WFA-med	7	40	451	830	1620	1481	50	1303	17680	42464	24874	15265	151	4082	n/a	n/a	n/a	n/a
WFA-low	7	30	308	554	884	960	39	828	10759	25321	12539	9852	114	2529	41819	52551	26067	19601
wfalm	5	125	2704	8968	26575	64781	136	12483	264104	898770	n/a	n/a	515	49181	n/a	n/a	n/a	n/a
wfalm-low	4	18	140	443	823	1426	20	535	4970	10435	30817	50766	58	1199	11658	36299	69312	958126
wfalm-rec	4	7	22	43	73	121	9	52	249	497	904	1445	15	106	549	1064	1787	n/a
BiWFA	9	10	13	19	27	35	26	32	66	97	180	229	46	64	122	202	267	378
BiWFA-score	7	8	9	16	23	32	17	29	64	97	186	223	35	56	122	204	256	350

Table S2: Execution time (s) and memory (MB) required per 1M bases aligned, using simulated sequences (100Kbp to 2Mbp).

4 Complementary evaluation on real data (shorter sequences)

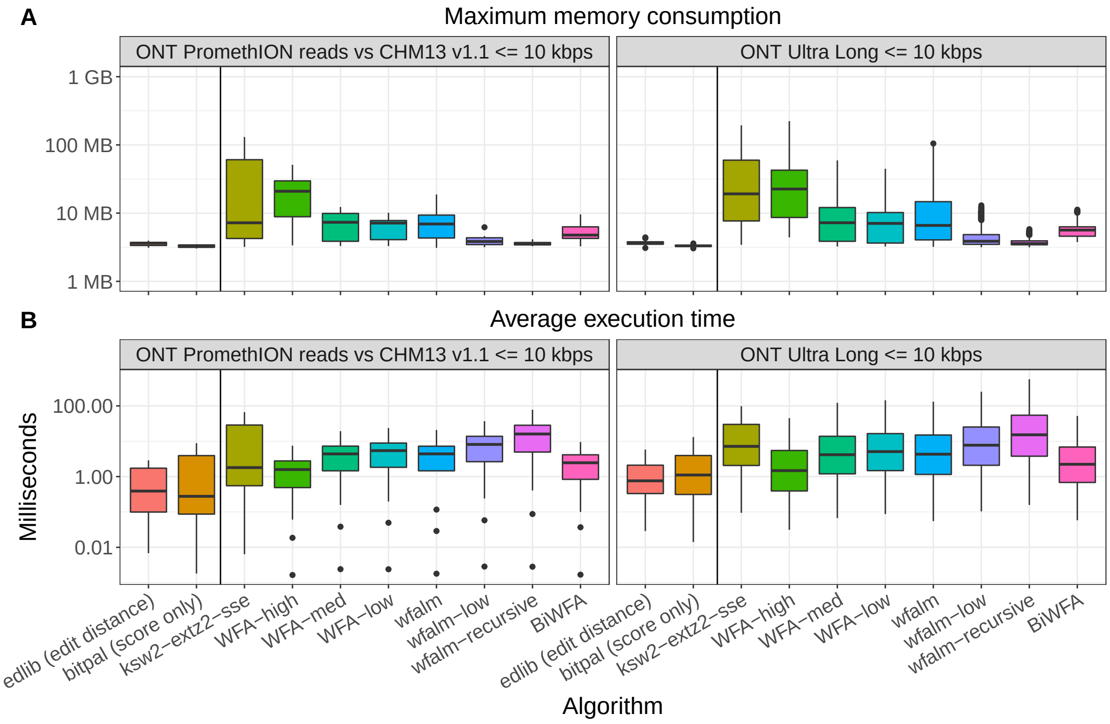

Figure S1: Experimental results from the execution of BiWFA and other state-of-the-art implementations aligning sequences up to 10Kbps. Figure shows (A) memory consumption and (B) execution time per sequence aligned. A vertical line on each panel separates algorithms that use simpler penalty models or can only compute the alignment score (i.e., edlib and bitpal) from those that compute the full gap-affine alignment.
